# Intracellular microRNA profiles form in the *Xenopus laevis* oocyte that may contribute to asymmetric cell division

**DOI:** 10.1038/srep11157

**Published:** 2015-06-10

**Authors:** Monika Sidova, Radek Sindelka, Mirco Castoldi, Vladimir Benes, Mikael Kubista

**Affiliations:** 1 Laboratory of Gene Expression, Institute of Biotechnology, Academy of Sciences of the Czech Republic, Videnska 1083, Prague, 142 20 Czech Republic; 2 Charles University in Prague, Faculty of Science, Department of Cell Biology, Vinicna 7, Prague, 128 43, Czech Republic; 3 Department of Gastroenterology Hepatology and Infectiology, University Hospital of Dusseldorf, Moorenstrasse 5, Dusseldorf, 40225 Germany; 4 EMBL Genomics Core Facility, Meyerhofstr. 1, Heidelberg, D-69117 Germany; 5 TATAA Biocenter AB, Odinsgatan 28, Göteborg, 411 03 Sweden

## Abstract

Asymmetric distribution of fate determinants within cells is an essential biological strategy to prepare them for asymmetric division. In this work we measure the intracellular distribution of 12 maternal microRNAs (miRNA) along the animal-vegetal axis of the *Xenopus laevis* oocyte using qPCR tomography. We find the miRNAs have distinct intracellular profiles that resemble two out of the three profiles we previously observed for mRNAs. Our results suggest that miRNAs in addition to proteins and mRNAs may have asymmetric distribution within the oocyte and may contribute to asymmetric cell division as cell fate determinants.

A central question in developmental biology is how the original fertilized oocyte gives rise to a complex organism made up of hundreds of different cell types. The key mechanism is asymmetric cell division that produces daughter cells with uneven distribution of deterministic biomolecules leading to different fates. Asymmetric cell division is induced by asymmetric cellular organization and polarization of fate determinants that are localized to distinct regions within the cell. Previously proteins and mRNA molecules have been identified as cell fate determinants [reviewed in^1^]. It has been shown that cell fate determinants are asymmetrically distributed already in the oocyte of several species, including the fruit fly (*Drosphila melanogaster*)^2^, the zebrafish (*Danio rerio*)^3^,^4^ and the African clawed frog (*Xenopus laevis*)^5^,^6^. This asymmetry leads to the formation of unequal blastomeres within the first few cell divisions^7^.

The *Xenopus* oocyte is an excellent model system to study early development. Its two distinguishable halves, referred to as the animal and vegetal hemispheres, are characterized by different pigmentation, which makes it easy to align the oocytes and study intracellular distributions of mRNAs. Most maternal mRNAs synthesized during oogenesis are evenly distributed along the animal-vegetal axis of *Xenopus* oocyte, though a small subset has been reported differentially distributed at either the animal or vegetal pole^8^,^9^. A more recent whole transcriptome analysis using microarrays suggest some 300 transcripts are localized in the vegetal cortex of the *Xenopus* oocytes^10^, which represents 2–3% of the maternally expressed genes. Two different pathways for the localization of the vegetal RNAs have been identified^11^. The early initiated pathway called METRO (messenger transport organizer) uses specific binding elements of mRNAs to attach them to a mitochondrial cloud that is formed during early oogenesis. Cis-acting elements, such as clusters of short nucleotide repeats, thought to mediate binding to a conserved complex of RNA-binding proteins, have been identified as well as trans-acting protein coding elements^12^. METRO is required for the localization of *cdx1* and *dazl* mRNAs that are incorporated into germinal granules needed for germ cell formation^13^. The late pathway is activated during mid and late oogenesis and depends on the cytoskeleton and microtubule motors^14^. It localizes particular mRNAs, such as *gdf1* and *vegt*, with sequence elements UUUCU and UUCAC^15^,^16^,^17^,^18^ in their 3’ untranslated regions (3´UTRs) that code for transcription factors controlling germ layer formation^19^. Notably, localized maternal mRNAs, such as *vegt, fatvg, xlsirts*, and *gdf1* in *Xenopus* and *oscar* in *Drosophila*, are important for the organization of cytokeratin and actin networks through a mechanism that does not require their translation^20^,^21^,^22^,^23^. *Vegt* mRNA, for example, is integrated into the cytoskeleton and its removal causes collapse of the cytokeratin network, while removal of *fatvg* mRNA induces hyperpolymerization of cytokeratin and actin filaments^20^,^24^. Some mRNAs have been reported more abundant in the animal hemisphere^25^. However, no particular mechanism has been described that would give rise to a localization of some mRNAs towards the animal pole and no particular functions have been ascribed to animally localized mRNA^1^,^8^. In fact, as we shall argue, weak polarization towards the animal side may develop spontaneously in the absence of active mechanisms and may in fact be the normal distribution. Another mechanism inducing intracellular rearrangement in the *Xenopus* oocyte is the cortical rotation. It follows fertilization by accumulation of dorsalizing biomolecules in the future dorsal side of the embryo and creates the dorsal-ventral developmental axis.

Using real-time quantitative PCR (qPCR) tomography and single cell expression profiling we measured intracellular mRNA profiles within the *Xenopus* oocyte and their distribution among blastomeres during the early developmental stages up to 32 cells. We discovered three distinct animal-vegetal mRNA profiles^26^, but found no evidence for mRNA asymmetry along the dorso-ventral or left-right axes^27^. We concluded that while the animal-vegetal asymmetry in *Xenopus* is induced by mRNA molecules the dorso-ventral asymmetry and possibly also the left-right asymmetry should be induced by uneven distribution of other biomolecules. Indeed, in several species asymmetric localization of β-catenin protein has been found critical for the formation of the dorso-ventral developmental axis^28^.

In this work we investigate if miRNAs show intracellular asymmetry, which would make them candidate cell fate determinants. miRNAs are 22–24 bases short single-stranded oligoribonucleotides that primarily regulate mRNA translation by binding to target mRNAs. Targeted locus of mRNAs for miRNA binding is usually at their 3’ UTR. It is well established that the 3’UTR of eukaryotic mRNAs is important for many of the mRNA functions. The 3’UTR site contains sequences that regulate the stability of the transcript, directs its localization, and may also contain other regulatory cis-acting elements. Recent studies have demonstrated that 3’UTRs can be functional independently of translation^29^. In this study we measure the intracellular profiles in mature *Xenopus* oocytes of the 12 selected miRNAs: miR-16c, miR-18b, miR-363-3p, miR-20b, miR-93a, miR-5102-5p, miR-19b, miR-221, miR-148b, miR-25, miR-22, miR-100, which have been ascribed maternal origin^30^,^31^, using qPCR tomography^26^,^32^.

## Results

We performed expression analysis of selected miRNAs and maternal mRNAs in *Xenopus laevis* oocytes using qPCR tomography^32^. The oocytes (in total six oocytes from two females) were cryo-sectioned along the animal-vegetal axis and extracted RNA was analyzed by RT-qPCR^26^,^32^. The intracellular distributions of the 12 maternal miRNAs: miR-16c, miR-18b, miR-363-3p, miR-20b, miR-93a, miR-5102-5p, miR-19b, miR-221, miR-148b, miR-25, miR-22 and miR-100 were measured along the animal-vegetal axis in five consecutive segments of the *Xenopus laevis* oocyte. In addition we measured the distributions of three mRNAs with known localizations: *maml1* (animal localization), *gdf1* (vegetal localization), and *cdx1* (extreme vegetal localization); reflecting the three mRNA animal-vegetal profiles found previously^26^,^27^. An RNA spike was used to optimize the extraction protocol. Measuring the RT-qPCR efficiency of the spike we found a maximum of 1.25 ng of total RNA could be used without compromising the reactions (Supplement Fig. 1).

**Figure 1:**
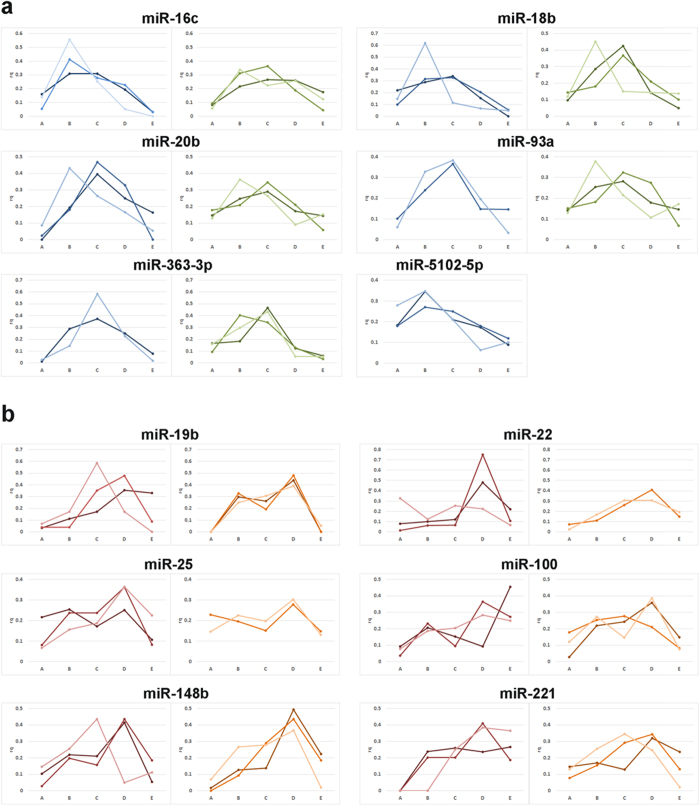
a. Individual intracellular profiles of miR-16c, miR-18b, miR-20b, miR-93a, miR-363-3p and miR-5102-5p predominantly localized in the center of the oocyte with slight asymmetry towards the animal hemisphere. Blue lines indicate oocytes from the first female and green lines indicate oocytes from the second female. **b**. Individual intracellular profiles of miR-19b, miR-22, miR-25, miR-100, miR-148b, and miR-221 localized in the vegetal hemisphere. Red lines indicate oocytes from the first female and orange lines indicate oocytes from the second female. Y-axis indicates relative quantity and x-axis indicates the section from the animal pole (section A) to vegetal pole (section E).

**Figure 2:**
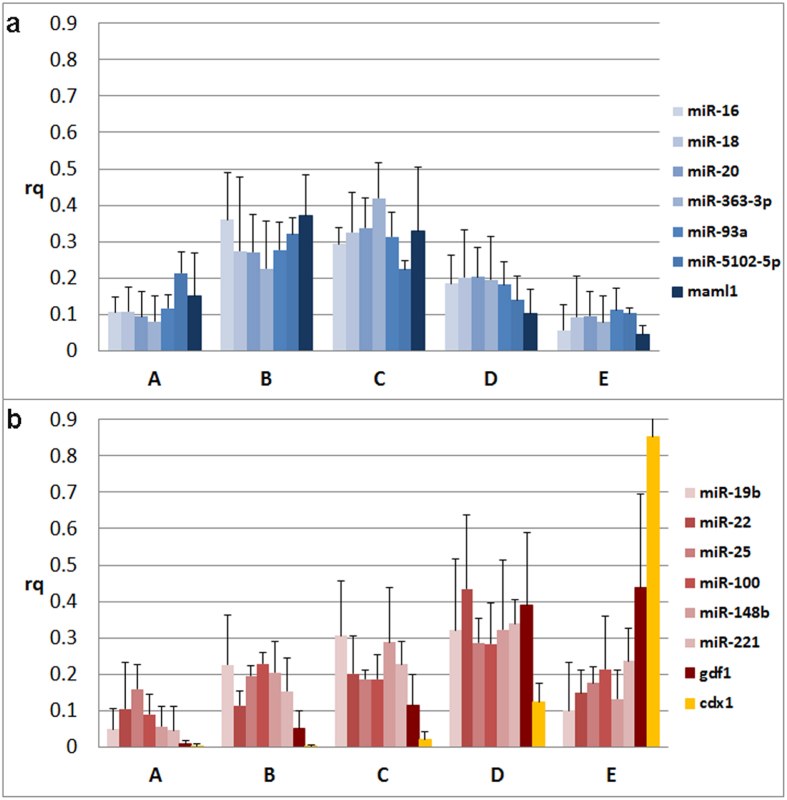
Intracellular profiles of the miRNAs and the selected reference mRNAs measured using qPCR tomography (*maml1*- dark blue, *gdf1* – dark red, *cdx1* - yellow). Y-axis indicates relative quantity and x-axis indicates the section from the animal pole (section A) to vegetal pole (section E). **a**. Spatial profiles of miR-16c, miR-18b, miR-20b, miR-93a, miR-363-3p and miR-5102-5p (indicated in blue scale), which are predominant in the animal hemisphere like the mRNA *maml1* (dark blue). **b**. Intracellular profiles of miR-19b, miR-22, miR-25, miR-100, miR-148b, and miR-221 (indicated in red scale), which are predominant in the vegetal hemisphere like the mRNA *gdf1* (dark red). Also shown is the mRNA *cdx1* (yellow), which has an extreme vegetal profile.

**Figure 3:**
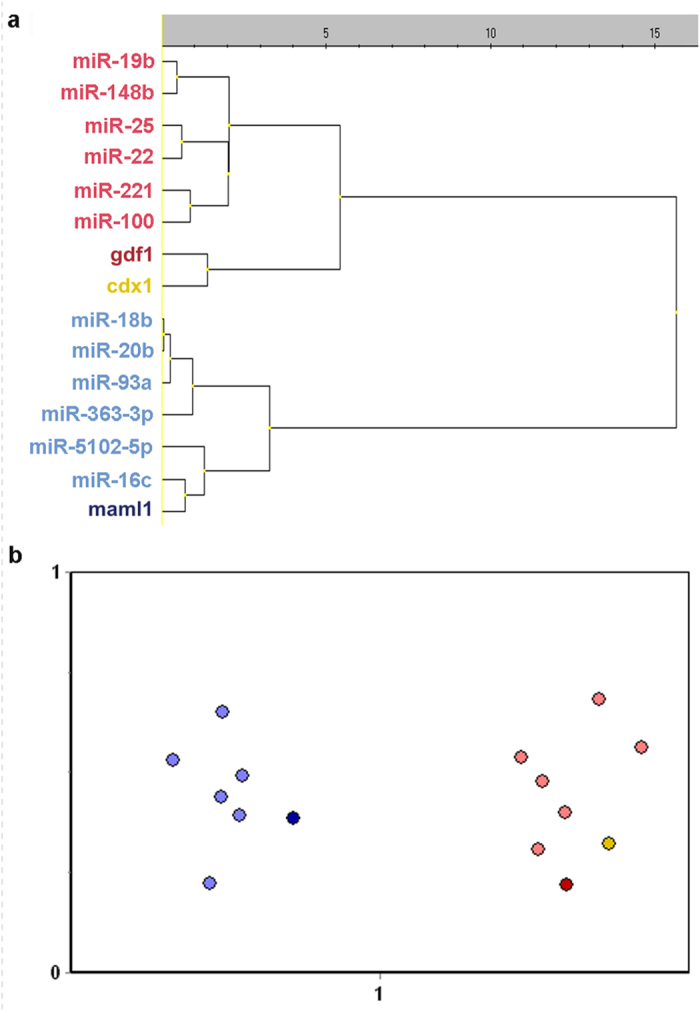
Hierarchical clustering (**a**) and SOM classification (**b**) of intracellular profiles along the animal-vegetal axis of the *Xenopus laevis* oocyte. The hierarchical clustering is presented in a dendrogram, where the similarity between miRNAs/mRNAs is indicated by the distance at which they are joined. Both the dendrogram and the SOM clearly separate the miRNA and mRNA profiles into two clusters. miR-16c, miR-18b, miR-20b, miR-93a, miR-363-3p and miR-5102-5p (light blue) cluster with animally localized mRNA *maml1* (dark blue), while miR-19b, miR-22, miR-25, miR-100, miR-148b, and miR-221 (light red) cluster with vegetally localized *gdf1* (dark red) and *cdx1* (yellow) mRNAs.

Our analysis revealed that the miRNAs we studied arrange in two distinct intracellular profiles that appear similar to two out of the three profiles we previously observed for mRNAs^26^. Specifically, miR-16c, miR-18b, miR-363-3p, miR-20b, miR-93a, miR-5102-5p and *maml1* mRNA are predominant in the center of the oocyte with slight asymmetry towards the animal hemisphere (Fig. 1a, Fig. 2a), while miR-19b, miR-221, miR-148b, miR-25, miR-22, miR-100 and *gdf1* mRNA are more abundant in the vegetal hemisphere (Fig. 1b, Fig. 2b). None of the studied miRNAs showed the extreme vegetal localization represented by the *cdx1* mRNA (Fig. 2b). Separation of the studied miRNAs into two groups with distinct animal-vegetal profiles was supported by the multivariate statistical analyses hierarchical clustering (presented as a dendrogram in Fig. 3a) and the Kohonen self-organizing map (SOM; Fig. 3b). Both classification methods clearly divide the miRNA and mRNA intracellular profiles into two clusters with preferential animal and vegetal distributions, respectively, and are henceforth referred to as the animal and vegetal groups.

**Figure 4:**
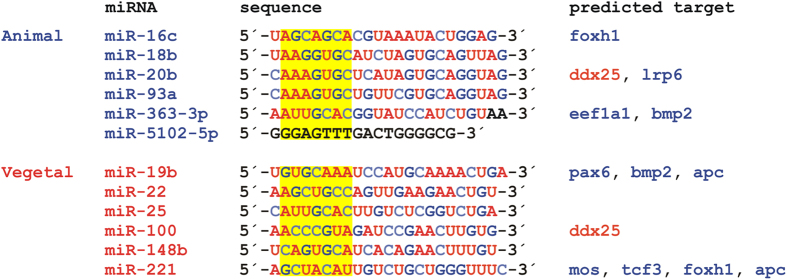
Sequences of the studied miRNAs and their predicted target mRNAs (animal mRNAs are shown in blue, vegetal mRNAs in red). Target prediction was performed using MicroCosm Targets Version 5 database (http://www.ebi.ac.uk/enright-srv/microcosm/htdocs/targets/v5/). The conservation is indicated by colored bases: red color indicates high evolutionary conservation, while blue color indicates low conservation. A sequence of miR-5102-5p indicated in black and two nucleotides on the 3´ end of miR-363-3p were not found in the database. Yellow boxes indicate miRNA seed sequences (from 2^nd^ to the 8^th^ nucleotide in the 5´ end).

We found no pattern within neither the animal nor the vegetal group that could be identified as a miRNA consensus sequence and ascribed to the asymmetric distribution of the miRNAs (Fig. 4). Using MicroCosm Targets Version 5 database (http://www.ebi.ac.uk/enright-srv/microcosm/htdocs/targets/v5/) we predicted target mRNAs for the studied miRNAs (Fig. 4) and were particularly interested in target mRNAs that form intracellular profiles along the animal-vegetal axis of the *Xenopus* oocyte^26^,^27^,^32^. The animal *foxh1*, *lrp6*, *eef1a1*, *bmp2*, *pax6*, *apc*, *mos* and *tcf3* mRNAs showed potential to hybridize with some of the studied miRNAs, but from both groups: animal miR-16c, miR-20b and miR-363-3p and vegetal miR-19b and miR-221. The only vegetal mRNA that was predicted to interact with asymmetrically localized miRNAs was *ddx25*. It showed complementarity to the animal miR-20b as well as to the vegetal miR-100.

## Discussion

Our results demonstrate that miRNAs, in addition to proteins and mRNAs, have asymmetric distribution in the *Xenopus* oocyte and may contribute to asymmetric cell division as cell fate determinants. Formation of intracellular profiles requires the cell offers an asymmetric environment. Indeed, the *Xenopus laevis* oocyte is asymmetric, with the animal and vegetal hemispheres having quite different compositions and the nucleus being located off center towards the animal pole^33^,^34^. There are also asymmetric perturbations, such as the sperm entry, that induce asymmetric distribution of cell fate determinants that leads to the dorsal-ventral asymmetry^19^. However, sperm entry does neither induce the mRNA asymmetry we reported earlier nor the miRNA asymmetry we observe here, as these profiles are present in the unfertilized oocyte.

miRNAs are transcribed in the cell nucleus and should stay close to it unless transported away. Since the nucleus in *Xenopus* oocytes is located closer to the animal hemisphere it is conceivable that passive diffusion of miRNAs secreted from the nucleus spontaneously produces the distribution with slight animal enrichment that we observe for half of the studied miRNAs (miR-16c, miR-18b, miR-20b, miR-93a, miR-363-3p and miR-5102-5p). These miRNAs could then reflect the normal population of miRNAs is not a word suitable to break apart. If really needed use: mi-RNAs. The distribution towards the vegetal hemisphere of the other miRNAs, however, must be driven by an active process. miRNAs bind their target mRNAs through hybridization requiring sequence complementarity. Although not all miRNA bases are required for sequence specific hybridization, there is hardly enough sequence flexibility to encompass a consensus binding sequence that could interact with one cellular component. We have extensively studied the sequences of the vegetally distributed miRNAs looking for patterns, but have not found motifs that could be identified as a consensus. Even though the gradient profiles along the animal-vegetal axis of miRNAs are similar to the profiles of mRNAs, we currently have no evidence that miRNA localization is induced by the binding of the miRNAs via a consensus sequence to a single localized molecular target. This raises the question how the miRNAs become asymmetrically distributed within the oocyte. Since interaction with an asymmetric environment is required, at least for the miRNAs with vegetal distribution, we find the idea that the vegetal miRNAs interact with vegetal mRNAs attractive. Despite extensive modelling with the MicroCosm Targets Version 5 database we have not been able to substantiate this hypothesis. This could be limitations in the *in silico* approach, which identifies sequences relevant for the transcriptional silencing mechanism mediated by miRNAs, which primarily is based on hybridization of complementary, consecutive bases, and does not consider more complex modes of interaction.

During our comparative analysis of miRNAs and search for consensus sequences, we also compared the seed sequences of the studied miRNAs. The seed sequence is a region from the 2^nd^ to the 8^th^ nucleotide at the 5´ end of the miRNA^35^. This sequence plays a critical role in the regulatory function of the miRNA through hybridization to the complementary 3´ UTR site of the target mRNA. The MicroCosm Targets Version 5 database predicts miRNA-mRNA interactions primarily based on sequence complementarity with highly rewarded seed sequence complementarity. We found that the three animally distributed miRNAs, miR-18b, miR-20b and miR-93a, have similar seed sequences: “AA_A_\^G^GUGC”. The database predicts that miR-20b (AAAGUGC) regulates *ddx25* and *lrp6* mRNAs, but does not identify those as targets for miR-18b, which has only one mismatch in the seed sequence (AAGGUGC). A single base mismatch in the seed sequence can perhaps be discriminative. However, the targets were not identified for miR-93a, which has identical seed sequence to miR-20b (AAAGUGC). Hence, the *in silico* analysis considers features beyond simple sequence complementarity, which are relevant for miRNA-mRNA interaction in relation to gene regulation, but may not apply to interactions leading to asymmetric intracellular distribution. It is conceivable these interactions are different, perhaps even involving non-consecutive sequences and exposed bases, and are therefore not recognized by the MicroCosm Targets Version 5 database. The numbers of known vegetally distributed mRNAs and in particular miRNAs are currently too few to develop models based on interactions that go beyond contiguous hybridization. As these numbers grow more advanced modelling will be possible. An interesting suggestion by an anonymous reviewer is that the miRNAs become actively distributed as pre-miRNAs, before being processed by Dicer, through sequences either in the loop or in the passenger strand. This is an attractive hypothesis and will be possible to test when sequence information about pre-miRNAs in *Xenopus laevis* becomes available. As for now the mechanism behind the vegetal distribution of miRNAs along the animal-vegetal axis within *Xenopus laevis* oocytes remains elusive.

In our studies of intracellular distribution of miRNAs and mRNAs we find molecules that co-localize with the nuclei and are more abundant in the animal hemisphere and we find molecules that show vegetal distribution. For miRNAs there is one vegetal profile, while for mRNAs there are two distinct vegetal profiles; one of which has extreme vegetal localization. In the literature many mRNAs with vegetal, extreme vegetal and animal distributions have been reported and in addition to those majority of mRNAs Is assumed to have normal distribution, which presumably should symmetric^8^. However, in our studies we have never found RNA molecules with even distribution across the oocyte. Of course, having only analyzed a rather small number of mRNAs and miRNAs those with even distribution may have escaped notice. Another possibility is there are no RNAs with even distribution across the oocyte; rather the distribution obtained in the absence of active mechanisms could have slight animal asymmetry due to the animal offset of the nuclei, where the RNAs are produced. We find this possibility attractive considering that no active mechanism behind animal distribution has ever been found and there is no evidence in the literature that the animal distribution should be different from normal. Hence, we propose mRNAs in the *Xenopus laevis* oocyte can have one out of three intracellular profiles: the spontaneous “normal” profile with slight localization towards the animal pole, vegetal localization, and extreme vegetal localization. For miRNAs we observe only the normal spontaneously formed animal profile and the vegetal localization.

In conclusion, we have discovered that certain miRNAs, including miR-16c, miR-18b, miR-363-3p, miR-20b, miR-93a and miR-5102-5p, are predominant in the animal hemisphere where the nuclei is located and possibly obtain this localization passively, while other miRNAs, including miR-19b, miR-221, miR-148b, miR-25, miR-22 and miR-100, are predominant in the vegetal hemisphere of the *Xenopus* oocyte. The latter miRNAs show similar internal profile as mRNAs oriented by the late pathway and should require active transport. None of the analyzed miRNAs displays the extreme vegetal distribution associated with the METRO oriented mRNAs. We speculate that the 3’UTR of some late pathway oriented mRNAs may contain sequences that bind the vegetally localized miRNAs or pre-miRNAs leading to co-localization. The vegetal distribution of some miRNAs suggests they contribute to asymmetric cell division and play a role in early embryogenesis.

## Methods

### Ethical Statement

This study was carried out in compliance with the Act No 246/1992 Coll., on the protection of animals against cruelty. Official permission was issued to the Faculty of Science; Charles University in Prague by the Central Commission for Animal Welfare of the Ministry of Agriculture of the Czech Republic (accreditation No. 24773/2008-10001, date of expiry 10.12.2013).

### *In vitro* fertilization, oocytes fixation and sectioning, RNA extraction

Two *Xenopus laevis* females were injected with 450 U of hCG (human chorionic gonadothropin) hormone and ovulated oocytes were obtained 12 hours after stimulation by gentle manual squeezing. Three oocytes from each female were collected and immediately embedded in a drop of OCT (optimal cutting temperature) medium on a pre-cooled dissection block. The block was placed for 10 minutes in the cryostat chamber (−19 °C) to temperature equilibrate the samples. The temperature of the section knife was −18 °C. The oocytes were cut into 45 slices, each being 30 μm thick, across the animal-vegetal axis. Consecutive slices were pooled into five tubes with nine slices in each. The first tube (denoted “A” in the figures) contained slices from the endmost animal part of the oocyte, while the last tube (denoted “E”) contains the endmost vegetal part. 300 μl of TRIzol reagent (Invitrogen) was added to each tube to extract total RNA. Samples were carefully homogenized by vortexing for 2 min and incubated for 5 min at room temperature to dissolve completely. Manufacturer’s instructions were followed during isolation, with the addition of 7 μg of glycogen (Sigma-Aldrich) to the isopropanol to enhance the RNA precipitation yield. Precipitated RNA was redissolved in 15 μl of RNase/DNase-free water and its concentration was measured using the Nanodrop ND1000 system.

### Control of inhibition

Yolk stored in the vegetal part of the oocyte is severe inhibitor of reverse transcription (RT) and quantitative PCR (qPCR). All samples were tested for inhibition using an RNA spike (TATAA Biocenter). SuperScript^TM^ III Reverse transcriptase kit (Invitrogen) was used for cDNA synthesis. Four different input amounts of total RNA (10 ng, 5 ng, 2.5 ng and 1.25 ng) were evaluated using RT-qPCR (Supplement Fig. 1). Severe inhibition was observed when analyzing the spike in the vegetal samples prepared from 10, 5 and 2.5 ng of total RNA. Using 1.25 ng of total RNA for cDNA synthesis the recovery yields of the RNA spike approached 100%. This was the same protocol as used in Flachsova *et al.* (2013)^27^. The cDNA samples were diluted 8 times to a final volume of 80 μl and stored at −20 °C. Three mRNAs (*maml1*, *cdx1* and *gdf1*), each representing one out of the three mRNA intracellular profiles observed previously^26^,^27^, were included for comparison. qPCR and melting curves were measured on a real-time CFX96 cycler system (BioRad). qPCR mix contained 2 μl of cDNA, 0.5 μl of forward and reverse primers (mixture 1:1, 10 μM each), 5 μl of iQ^TM^ SYBR® Green Supermix (Bio-Rad), and deionized water in a total volume of 10 μl. The temperature profile was: activation of polymerase at 95 °C for 3 min., followed by 40 cycles of denaturation at 95 °C for 15 sec., annealing at 60 °C for 20 sec., and elongation at 72 °C for 30 sec. The formation of expected PCR products was confirmed by measuring melting curves between 65 °C and 95 °C in 0.5 °C intervals.

### Quantification of miRNA by qPCR

miRNA expression profiling was performed using miQPCR^36^, which is a method for global RT-qPCR profiling of miRNAs^37^,^38^. Briefly, the 3’-ends of single-stranded RNAs are extended uniformly with a specific adaptor named miLINKER. This adapter is then used as an anchor to prime cDNA synthesis during reverse transcription and for detection of the selected amplicon during the qPCR. For each sample 1.25 ng of total RNA was treated with the miLINKER (5 μM for each extension). Elongated samples were reverse transcribed using Superscript II (Invitrogen) following the supplier’s instructions. miRNA specific qPCR requires 100 pg of cDNA, 2.5 μM of the universal primer (i.e. complementary to the miLINKER) and 2.5 μM of a miRNA specific primer. qPCR of miRNAs was performed using iQ^TM^ SYBR® Green Supermix (Bio-Rad, total reaction volume 10 μl) on an CFX384 cycler (BioRad) with the same protocol as for the mRNAs described above. Tailing, reverse transcription and qPCR reagents were prepared as mastermixes for all 30 samples (six oocytes; each 5 sections). The miRNA specific primers were designed to be complementary to the target miRNA with a G nucleotide overhang on the 3´end, which binds to the first nucleotide of the attached miLINKER. The annealing temperature was set to 60 °C and estimated using the Tm calculator provided by Applied Biosystems (http://www6.appliedbiosystems.com/support/techtools/calc/). Primer specificity was verified by blast (http://blast.ncbi.nlm.nih.gov/Blast.cgi). Primer assays were designed for 27 selected maternal miRNAs. 12 assays with highest efficiency (probably the most abundant) and robustness were used for expression profiling (Supplement Tab. 1).

### Data analysis

The measured Cq values of the animal-vegetal segments were converted to relative quantities using the equation (1): 
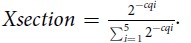
. Where Cq_i_ is the Cq value measured in the i^th^ section. The same amount of total RNA was analyzed per section serving as normalizer. x_section_ can thus be considered as the relative amount or fraction of the particular miRNA/mRNA in that segment^32^. The relative amounts in each segment were averaged across all oocytes from both females and are presented in bar/line charts with the x-axis representing the segment position from the animal pole along the animal-vegetal axis and the y-axis indicating the relative quantity (Fig. 1, Fig. 2). Individual profiles of all 12 miRNAs can be found in Fig. 1. The data were also analyzed with the software GenEx (MultiD) to determine correlations between the miRNAs’ and maternal mRNAs’ intracellular profiles. For multivariate analysis data were autoscaled along genes and profiles were analyzed for similarities using the Kohonen self-organizing map (SOM) and hierarchical clustering (Fig. 3). We used SOM with two boxes (2 × 1) to force the data into two groups based on the measured profiles. Using three boxes, one box ended up empty supporting the conclusion that the profiles indeed reflect only two distinct distributions (not shown). Independent SOM trainings yielded the same two groups evidencing robust classification. SOM training of only the miRNAs, leaving out the mRNAs, gave a separation into the same groups, evidencing that the separation is not induced by the mRNAs but a property of the miRNAs. The biological variability between the females was tested using unpaired 2-tails t-test. The p-values were > 0.05 indicating higher variability among oocytes than between samples from different females.

All methods were carried out in accordance with the approved guidelines and all experimental protocols were approved by named institutions, including any relevant details in the methods section.

## Additional Information

**How to cite this article**: Sidova, M. *et al.* Intracellular microRNA profiles form in the *Xenopus laevis* oocyte that may contribute to asymmetric cell division. *Sci. Rep.* **5**, 11157; doi: 10.1038/srep11157 (2015).

## Supplementary Material

Supplementary Information

## References

[b1] Houston D. W. Regulation of cell polarity and RNA localization in vertebrate oocyte. Int Rev Cell Mol Biol. 306, 127–185 (2013). 24016525 10.1016/B978-0-12-407694-5.00004-3

[b2] Ephrussi A. & Lehmann R. Induction of germ cell formation by oskar. Nature 358, 387–392 (1992). 1641021 10.1038/358387a0

[b3] Hashimoto Y. *et al.* Localized maternal factors are required for zebrafish germ cell formation. Dev. Biol. 268, 152–161 (2004). 15031112 10.1016/j.ydbio.2003.12.013

[b4] Howley C. & Ho R. K. mRNA localization patterns in zebrafish oocytes. Mech. Dev. 92, 305–309 (2000). 10727871 10.1016/s0925-4773(00)00247-1

[b5] Kloc M. & Etkin L. D. RNA localization mechanisms in oocytes. J Cell Sci. 118, 269–282 (2005). 15654016 10.1242/jcs.01637

[b6] King M. L., Zhou Y. & Bubunenko M. Polarizing genetic information in the egg: RNA localization in the frog oocyte. Bioessays 21, 546–557 (1999). 10472182 10.1002/(SICI)1521-1878(199907)21:7<546::AID-BIES3>3.0.CO;2-Z

[b7] Pandur P. D., Sullivan S. A. & Moody S. A. Multiple maternal influences on dorsal-ventral fate of Xenopus animal blastomeres. Dev Dyn. 225, 581–587 (2002). 12454934 10.1002/dvdy.10181

[b8] King M. L., Messitt T. J. & Mowry K. L. Putting RNAs in the right place at the right time: RNA localization in the frog oocyte. Biol Cell. 97, 19–33 (2005). 15601255 10.1042/BC20040067

[b9] Mowry K. L. & Cote C. A. RNA sorting in Xenopus oocytes and embryos. FASEB J. 13, 435–445 (1999). 10064610 10.1096/fasebj.13.3.435

[b10] Cuykendall T. N. & Houston D. W. Identification of germ plasm-associated transcripts by microarray analysis of Xenopus vegetal cortex RNA. Dev Dyn. 239, 1838–1848 (2010). 20503379 10.1002/dvdy.22304 PMC3065113

[b11] Kloc M. & Etkin L. D. Two distinct pathways for the localization of RNAs at the vegetal cortex in Xenopus oocytes. Development 121, 287–297 (1995). 7539356 10.1242/dev.121.2.287

[b12] Snedden D. D., Bertke M. M., Vernon D. & Huber P. W. RNA localization in Xenopus oocytes uses a core group of trans-acting factors irrespective of destination. RNA 19, 889–895 (2013). 23645708 10.1261/rna.038232.113 PMC3683923

[b13] Zhou Y. & King M. L. Sending RNAs into the future: RNA localization and germ cell fate. IUBMB Life 56, 19–27 (2004). 14992376 10.1080/15216540310001658886

[b14] Yisraeli J. K., Sokol S. & Melton D. A. A two-step model for the localization of maternal mRNA in Xenopus oocytes: involvement of microtubules and microfilaments in the translocation and anchoring of Vg1 mRNA. Development 108, 289–298 (1990). 2351071 10.1242/dev.108.2.289

[b15] Deshler J. O., Highett M. I. & Schnapp B. J. Localization of Xenopus Vg1 mRNA by Vera protein and the endoplasmic reticulum. Science 276, 1128–1131 (1997). 9148809 10.1126/science.276.5315.1128

[b16] Gautreau D., Cote C. A. & Mowry K. L. Two copies of a subelement from the Vg1 RNA localization sequence are sufficient to direct vegetal localization in Xenopus oocytes. Development 124, 5013–5020 (1997). 9362462 10.1242/dev.124.24.5013

[b17] Lewis R. A. *et al.* Conserved and clustered RNA recognition sequences are a critical feature of signals directing RNA localization in Xenopus oocytes. Mech Dev. 121, 101–109 (2004). 14706704 10.1016/j.mod.2003.09.009

[b18] Bubunenko M., Kress T. L., Vempati U. D., Mowry K. L. & King M. L. A consensus RNA signal that directs germ layer determinants to the vegetal cortex of Xenopus oocytes. Dev Biol. 248, 82–92 (2002). 12142022 10.1006/dbio.2002.0719

[b19] White J. A. & Heasman J. Maternal control of pattern formation in Xenopus laevis. J Exp Zool B Mol Dev Evol. 310, 73–84 (2008). 17219372 10.1002/jez.b.21153

[b20] Kloc M., Bilinski S. & Dougherty M. T. Organization of cytokeratin cytoskeleton and germ plasm in the vegetal cortex of Xenopus laevis oocytes depends on coding and non-coding RNAs: three-dimensional and ultrastructural analysis. Exp Cell Res. 313, 1639–1651 (2007). 17376434 10.1016/j.yexcr.2007.02.018 PMC2613015

[b21] Kloc M. Teachings from the egg: new and unexpected functions of RNAs. Mol Reprod Dev. 76, 922–932 (2009). 19422043 10.1002/mrd.21043

[b22] Kloc M., Foreman V. & Reddy S. A. Binary function of mRNA. Biochimie 93, 1955–1961 (2011). 21784124 10.1016/j.biochi.2011.07.008

[b23] Jenny A. *et al.* A translation-independent role of oskar RNA in early Drosophila oogenesis. Development 133, 2827–2833 (2006). 16835436 10.1242/dev.02456

[b24] Kloc M. *et al.* Potential structural role of non-coding and coding RNAs in the organization of the cytoskeleton at the vegetal cortex of Xenopus oocytes. Development 132, 3445–3457 (2005). 16000384 10.1242/dev.01919

[b25] Kloc M. *et al.* RNA localization and germ cell determination in Xenopus. Int Rev Cytol. 203, 63–91 (2001). 11131528 10.1016/s0074-7696(01)03004-2

[b26] Sindelka R., Sidova M., Svec D. & Kubista M. Spatial expression profiles in the Xenopus laevis oocytes measured with qPCR tomography. Methods 51, 87–91 (2010). 20051264 10.1016/j.ymeth.2009.12.011

[b27] Flachsova M., Sindelka R. & Kubista M. Single blastomere expression profiling of Xenopus laevis embryos of 8 to 32-cells reveals developmental asymmetry. Sci Rep 3, 2278 (2013). 23880666 10.1038/srep02278 PMC3721081

[b28] Wylie C. *et al.* Maternal beta-catenin establishes a 'dorsal signal' in early Xenopus embryos. Development 122, 2987–2996 (1996). 8898213 10.1242/dev.122.10.2987

[b29] Mercer T. R. *et al.* Expression of distinct RNAs from 3' untranslated regions. Nucleic Acids Res. 39, 2393–2403 (2011). 21075793 10.1093/nar/gkq1158 PMC3064787

[b30] Watanabe T. *et al.* Stage-specific expression of microRNAs during Xenopus development. FEBS Lett. 579, 318–324 (2005). 15642338 10.1016/j.febslet.2004.11.067

[b31] Ambady S., Wu Z. & Dominko T. Identification of novel miRNAs in Xenopus laevis matephase II arrested eggs. Genesis 50, 286–299 (2012). 22223599 10.1002/dvg.22010 PMC3309558

[b32] Sindelka R., Jonak J., Hands R., Bustin S. A. & Kubista M. Intracellular expression profiles measured by real-time PCR tomography in the Xenopus laevis oocyte. Nucleic Acids Res. 36, 387–392 (2008). 18039714 10.1093/nar/gkm1024 PMC2241880

[b33] Gurdon J. B. Changes in somatic cell nuclei inserted into growing and maturing amphibian oocytes. J Embryol Exp Morph. 20, 401–414 (1968). 5750105

[b34] Jullien J., Pasque V., Halley-Stott R. P., Miyamoto K. & Gurdon J. B. Mechanism of nuclear reprogramming by eggs and oocytes: a deterministic process? Nat Rev Mol Cell Biol. 12, 453–459 (2011). 21697902 10.1038/nrm3140 PMC3657683

[b35] Hibio N., Hino K., Shimizu E., Nagata Y. & Ui-Tei K. Stability of miRNA 5´terminal and seed regions in correlated with experimentally observed miRNA-mediated silencing efficacy. Sci Rep. 2, 996 (2012). 23251782 10.1038/srep00996 PMC3524778

[b36] Benes V. *et al.* Identification of cytokines-induced modulation of microRNAs expression and secretion as measured by a novel microRNA specific qPCR assay. Accepted in Sci Rep. (2015). 10.1038/srep11590 PMC4480321 26108880

[b37] Ibberson D., Benes V., Muckenthaler M. U. & Castoldi M. RNA degradation compromises the reliability of microRNA expression profiling. BMC Biotechnol. 9, 102 (2009). 20025722 10.1186/1472-6750-9-102 PMC2805631

[b38] Castoldi M. *et al.* The liver-specific microRNA miR-122 controls systemic iron homeostasis in mice. J Clin Invest. 121, 1386–1396 (2011). 21364282 10.1172/JCI44883 PMC3069782

