# Supplementary material for: Intracellular microRNA profiles form in the Xenopus laevis oocyte that may contribute to asymmetric cell division
Source: Sci Rep. 2015 Jun 10;5:11157. doi: 10.1038/srep11157 (PMC4461913; doi:10.1038/srep11157)
Supplement: Supplementary Information [file srep11157-s1.pdf]

Intracellular microRNA profiles form in the *Xenopus laevis* oocyte that may contribute to asymmetric cell division

Authors

Monika Sidova, Radek Sindelka, Mirco Castoldi, Vladimir Benes and Mikael Kubista

Supplement information

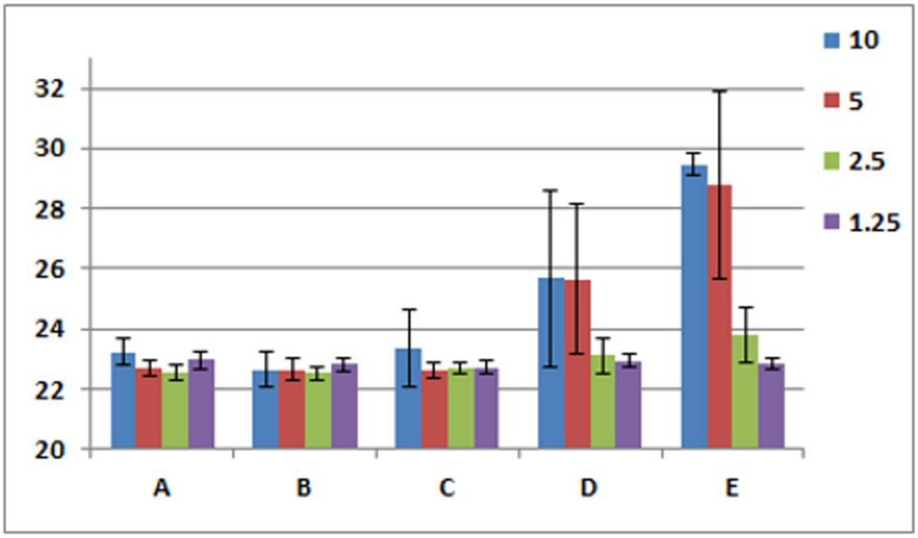

Supplement Figure 1. Optimization of experimental protocol using an RNA spike. 10 ng (blue), 5 ng (red), 2.5 ng (green), and 1.25 ng (violet) of total RNA and same amount of the RNA spike were used for reverse transcription. Spike cDNA was than quantified using qPCR. Y-axis shows Cq value and x-axis indicates section from the animal pole (section A) to vegetal pole (section E). Error bars indicate standard deviation of biological replicates.

miRNA	primer	Tm
miR-16c	TAGCAGCACGTAAATACTGGAGG	59.30
miR-18b	TAAGGTGCATCTAGTGCAGTTAGG	59.52
miR-19b	TGCAAATCCATGCAAAACTGAG	61.83
miR-20b	CAAAGTGCTCATAGTGCAGGTAGG	60.98
miR-22	AGCTGCCAGTTGAAGAACTGTG	60.44
miR-25	ATTGCACTTGTCTCGGTCTGAG	60.27
miR-93a	GTGCTGTTCGTGCAGGTAGG	60.29
miR-100	CCGTAGATCCGAACTTGTGG	58.99
miR-148b	TCAGTGCATCACAGAACTTTGTG	59.79
miR-221	GCTACATTGTCTGCTGGGTTTG	60.50
miR-363-3p	AATTGCACGGTATCCATCTGTAAG	60.08
miR-5102-5p	GAGTTTGACTGGGGCGG	58.52
miR-15a	TAGCAGCACATAATGGTTTGTGAG	60.02
miR-15c	TAGCAGCACATCATGGTTTGTAG	58.77
miR-22	AGCTGCCAGTTGAAGAACTGTG	60.44
miR-24a	GCTCAGTTCAGCAGGAACAGG	60.93
miR-27b	TCACAGTGGCTAAGTTCTGCG	60.03
miR-34a	GGCAGTGTCTTAGCTGGTTGTG	60.69
miR-34b	AGGCAGTGTAGTTAGCTGATTGG	58.47
miR-122	TGGAGTGTGACAATGGTGTTTG	60.28
miR-124	ACGCGGTGAATGCCAAG	60.20
miR-140-3p	CACAGGGTAGAACCACGGAG	59.00
miR-191	GAATCCCAAAAGCAGCTGTG	59.26
miR-210	CGTGTGACAGCGGCTGAG	61.23
miR-214	CAGGCACAGACAGGCAGTG	60.03
miR-222	ACATCTGGCTACTGGGTCTCG	60.11
miR-375	CGTTCGGCTCGCGTTAG	60.08

Supplement Table 1. Primer assays and their predicted Tm of maternal miRNAs. The primer assays were designed for 27 randomly selected maternal miRNAs. 12 assays (miR-16c, miR-18b, miR-19b, miR-20b, miR-22, miR-25, miR-93a, miR-100, miR-148b, miR-221, miR-363-3p, miR-5102-5p, shown in red) showed higher and more reproducible expression than the other 15 miRNAs (miR-15a, miR-15c, miR-22, miR-24a, miR-27b, miR-34a, miR-34b, miR-122, miR-124, miR-140-3p, miR-191, miR-210, miR-214, miR-222, miR-375, shown in blue).
